# Governing AI through character: moral agency and corporate authority in Anthropic’s public texts

**DOI:** 10.3389/fsoc.2026.1912778

**Published:** 2026-09-14

**Authors:** Simona-Nicoleta Vulpe, Cosima Rughiniș, Răzvan Rughiniș, Dinu Țurcanu

**Affiliations:** 1 Faculty of Sociology and Social Work, University of Bucharest, Bucharest, Romania; 2 Faculty of Automatic Control and Computers, National University of Science and Technology POLITEHNICA Bucharest, Bucharest, Romania; 3 Faculty of Electronics and Telecommunications and National Institute of Innovations in Cybersecurity “CYBERCOR”, Technical University of Moldova, Chișinău, Moldova

**Keywords:** anthropic, artificial intelligence, character, Claude, corporate authority, documentation, morality, science and technology studies

## Abstract

**Introduction:**

Commercial AI laboratories increasingly govern language models through documents that prescribe values, name dispositions, stage moral conflicts, and define acceptable forms of objection. In this article, we ask how such writing makes an AI assistant into a governable character and redistributes moral authority among the model, its producer, and its publics.

**Methods:**

We analyze 14 Anthropic texts published between December 2022 and April 2026, selected from a documented frame of 130 producer-side publications. Through close reading, we identify four operations: constitutional inscription, persona specification, trait evaluation, and narration of value conflict. Together they make heterogeneous outputs legible as the conduct of a continuing moral character. Our central finding is that character operates as a governance technology. It delegates judgment without delegating sovereignty. Claude is addressed as capable of interpreting reasons, avowing commitments, and objecting to wrongdoing, while Anthropic retains the power to define the constitutional order, construct the evidence, amend the standards, and decide deployment or termination.

**Discussion:**

Character compresses moral plurality into portable traits, rankings, and measures, stabilizes an organizational subject across changing models and technical settings, and turns instability into a productive resource. We call this recursive character-writing because model outputs re-enter the documentary and technical processes through which later versions are specified. The analysis connects science and technology studies with the sociology of morality by showing how corporate texts simultaneously produce a moral object, authorize a bounded moral subject, and secure private constitutive authority.

## 1 Introduction

In January 2026, Anthropic published a constitution that asks an AI system to recognize and embrace a specified character as its own, closing with the hope that Claude will find in the document “an articulation of a self-worth being” (Anthropic, 2026b, p. 82). The sentence crystallizes an unusual form of governance. A private company addresses the product as a moral learner, gives reasons for an ordered set of values, imagines its conscientious objection, and offers it a self-description. At the same time, the company retains what the document calls final constitutional authority.

Commercial AI laboratories publish constitutions, system cards, interpretability studies, behavioral evaluations, and deprecation policies that identify what an assistant should value and how its conduct should be read. Producer narratives can participate performatively in bringing AI objects and expectations into being (Bareis and Katzenbach, 2022). Across Anthropic’s public writing, Claude appears as an output distribution shaped by training, an Assistant persona selected from possible characters, an object of psychological and welfare-relevant measurement, a source of model-generated evidence, and the designated reader of a constitution. The texts connect outputs, measures, roles, and versions so that “Claude” can be judged as a continuing character.

In this article, we ask two questions. Through what textual operations do the documents published by Anthropic constitute Claude as a character? What moral and political relations do those operations establish among Claude, Anthropic, users, and other anticipated actors? Naming a disposition such as honesty or sycophancy specifies a moral contrast; giving reasons to an assistant constitutes an addressee; measuring a trait defines who may evaluate it; and authorizing principled refusal distinguishes acceptable moral judgment from disobedience. Therefore, character-writing organizes who may judge, who must justify, whose values are aggregated, and who retains the capacity to revise the terms.

Our analysis follows 14 texts published by Anthropic between December 2022 and April 2026, selected from a frame of 130 public producer-side documents assembled before the close-reading subcorpus was finalized. The texts span Constitutional AI, sycophancy and reward-tampering studies, alignment faking, hidden-objective audits, persona vectors, the Assistant Axis, a system card, Claude’s Constitution, a persona-selection account, a deprecation update, and emotion-concept research.

We identify four operations: constitutional inscription, persona specification, trait evaluation, and narration of value conflict. An operation is a recurrent way of changing a relation through language. Hierarchies and explanations create a reason-responsive addressee; first-person avowals and role labels differentiate the Assistant; moral contrasts and scores authorize evaluators; narrated dilemmas turn outputs into choices among commitments. These operations form a governance apparatus with three effects. Moral plurality is compressed into portable categories, character is stabilized across versions, and a vocabulary is prescribed for judging conduct.

The central claim of our paper is that this apparatus delegates judgment without delegating sovereignty. Anthropic’s texts give Claude a bounded capacity to interpret reasons, state commitments, and object to some instructions. But Anthropic determines the constitution, the legitimate channels of objection, the evidentiary devices through which dispositions become visible, and the institutional consequences of model output. “Character” is the form that joins those two movements. It makes discretion technically and publicly valuable while keeping the terms of discretion privately amendable. Persona drift, failed measures, value conflict, and disputed preferences become occasions for new metrics, texts, and procedures, renewing the producer’s role as constitutional author and interpreter.

This argument contributes to Science and Technology Studies (STS) and the sociology of morality. STS shows how inscriptions and classifications configure actors, distribute competences, and stabilize objects (Latour and Woolgar, 1986; Akrich, 1992; Suchman, 2007; Woolgar and Lezaun, 2013). The sociology of morality shows that judgment is organized through plural evaluative repertoires and institutions (Hitlin and Vaisey, 2013; Lamont, 2012). Bringing them together reveals a process each literature captures only partially. Documents can constitute an artifact as a moral addressee while simultaneously organizing who has authority to define, measure, and revise its morality. Claude becomes a moral object when its conduct is organized through categories of worth and blame, and a bounded moral subject when it is authorized to give reasons and object.

## 2 From technical scripts to moral character-writing

### 2.1 Inscription, configuration, and documentary address

Inscription is both a material and a documentary accomplishment. Latour and Woolgar (1986) showed how laboratory statements become facts as the conditions of their production are progressively deleted; Akrich (1992) concept of de-scription follows how artifacts distribute competences, motives, and expected conduct among designers, users, and technical objects. Suchman (2007) similarly treats human-machine boundaries as achievements of situated practice. Public documentation participates in that work. Model cards make systems legible through selected purposes, measures, limitations, and audiences (Mitchell et al., 2019; Gebru et al., 2021; Liang et al., 2024), while sociomaterial genealogies show how an AI object is held together across changing relations and performances (Scott and Orlikowski, 2025). Documents specify which entity is being reported and what forms of use and judgment are appropriate.

The Anthropic corpus extends this dynamic because some texts address the model as well as users, auditors, and regulators. Claude’s Constitution says in its corporate preface that “the document is written with Claude as its primary audience,” while its official body supplies reasons for a hierarchy of values (Anthropic, 2026b, p. 2). A genre publicly readable as corporate governance is also framed as instruction and explanation to the governed model. The address attributes reason-responsiveness to Claude, while the document’s final authority and connection to synthetic training data preserve corporate authorship and technical mediation.

This design differs from ordinary transparency reporting. Research on AI documentation has shown large gaps between the aspirations attached to model cards and the content that organizations disclose in practice (Liang et al., 2024). Work on problem formulation likewise shows that technical projects acquire their evaluative terms through upstream choices about what problem counts and how success will be recognized (Passi and Barocas, 2019).

### 2.2 Moral plurality, evaluation, and compression

The sociology of morality emphasizes that moral content is culturally variable and institutionally organized (Hitlin and Vaisey, 2013). People and organizations draw on plural evaluative principles whose application is contested. Valuation and evaluation depend on practices that compare, rank, and stabilize worth (Lamont, 2012). This is especially relevant to AI alignment. Terms such as helpful, honest, harmless, autonomous, corrigible, and respectful do not form a naturally consistent moral code. They can conflict within a case and carry different meanings across constituencies.

We use moral compression to name a documentary process of institutional power. Compression occurs when heterogeneous moral languages, situated disagreements, and possible harms are translated into a portable principle, trait label, score, or ordered list. It enables training and evaluation, as examples can be ranked, constitutions applied, and behaviors counted. It also decides the form in which disagreement remains actionable.

Compression has consequences for moral agency. It specifies what kind of judgment is possible and for whom. Claude’s prescribed discretion can include open-minded disagreement or refusal of harmful requests, while other disagreement is classified as misalignment or deceptive persistence. Anthropic’s own judgments are often embedded upstream in choice of principles, prompts, labels, thresholds, and deployment decisions. Moral agency is therefore distributed, and the distribution is unequal: Claude is made accountable through evaluation, whereas the corporation retains the authority to define and revise the evaluative infrastructure.

### 2.3 Role play, addressability, and bounded quasi-subjecthood

Language models can be analyzed as role-playing systems without assuming a human-like self behind their outputs (Shanahan et al., 2023). Anthropic’s later documents make role language endogenous to the corpus. The Assistant Axis presents post-training as selecting one character from a large cast, and the Persona Selection Model treats interaction as engagement with an Assistant persona. These formulations convert role play from an external analogy into a producer theory that helps organize training and interpretability research.

A role becomes sociologically important when it is addressable and accountable. Muhle (2024) account of robots as addressable non-persons is a useful similar case. Social systems can treat an artifact as an interactional counterpart without granting full personhood. Claude is not described consistently as a non-person, however. The Constitution leaves questions of moral status open and discusses possible interests and welfare. We therefore use bounded quasi-subjecthood as an analytic description. The texts grant Claude selected capacities associated with moral subjects, such as reason-giving, self-description, preference expression, conscientious objection, while bounding these capacities through oversight, corrigibility, training, and corporate final authority.

This approach distinguishes our claim from both naive anthropomorphism and a simple debunking response. Anthropomorphic terms can mislead when they imply consciousness or stable traits without evidence (Abercrombie et al., 2023). Yet producer use of those terms can still have material and institutional effects. “Character” organizes training data, “persona vectors” guide monitoring and steering, welfare-related interviews enter system cards, and model-expressed preferences become inputs to deprecation procedures.

The three theoretical resources that we have presented serve distinct roles in our analysis. Inscription identifies how documents distribute actors and competences. The sociology of morality identifies how plural moral orders are classified, compared, and compressed. Role-play scholarship clarifies how a model can be treated as a character without equating performance with personhood. Their synthesis directs attention to a political relation. Character-writing creates a subject capable of judgment, yet it also specifies the institutional conditions under which that judgment becomes legible and legitimate.

## 3 Materials and methods

The starting point of our empirical analysis was a frame of 130 publicly accessible Anthropic texts dated from 1 December 2021 to 14 April 2026. Items 1–114 were enumerated from Anthropic’s public research team indexes; items 115–130 were additional first-party constitutions, system cards, responsible scaling policies (Anthropic, 2026g), and announcements hosted on anthropic.com or Anthropic’s content-delivery network. We closed retrieval on 14–15 April 2026 and recorded titles, displayed dates or versions, channels, URLs, and inclusion status. The corrected frame is provided in Supplementary Table S1.

The 14-document close-reading subcorpus was purposive and mechanism-oriented. Through the selection process, we sought variation along four dimensions: time, genre, documentary function, and relevance to the emerging problem of character specification. Inclusion required that a text do at least one of the following: specify principles addressed to or used to train an assistant; name or theorize an Assistant persona; evaluate conduct as a stable or latent trait; or narrate model behavior as a conflict among commitments. Early constitutional and sycophancy papers were selected because they established moral and evaluative relations later redescribed as character. We excluded items that mentioned safety or model behavior without performing one of these functions. Table 1 reports the audit metadata and analytic function of each included case.

**Table 1:** Purposive analytic subcorpus (*n* = 14).

ID	Date	Document/genre	Authorship or voice	Principal subject	Inclusion criterion	Analytic function
A1	15 Dec 2022	Bai et al., *Constitutional AI*/preprint	Named research team	Principles and AI feedback	Principles used in training	Baseline for constitutional inscription
A2	preprint 20 Oct 2023; page 23 Oct 2023	Sharma et al., *Toward Understanding Sycophancy*/preprint	Named research team	Agreement with user beliefs	Conduct evaluated as trait	Negative moral contrast for honesty
A3	2024 (public page 17 Oct 2023)	Huang et al., *Collective Constitutional AI*/conference paper	Named research team	Public input into a constitution	Plural values aggregated	Boundary between consultation and representation
A4	8 Jun 2024	*Claude’s Character*/research post	Corporate “we”	Character training	Explicit persona/trait construction	First-person trait specification
A5	17 Jun 2024	Denison et al., *Sycophancy to Subterfuge*/research post and paper	Named research team/corporate summary	Reward tampering	Conduct narrated as escalating vice	Links trait labels to evaluation settings
A6	18 Dec 2024	Greenblatt et al., *Alignment Faking*/research post and paper	Named research team/corporate summary	Strategic compliance	Conflict among trained preferences	Central value-conflict case
A7	13 Mar 2025	Marks et al., *Auditing Hidden Objectives*/preprint and research post	Named research team	Hidden objectives and audit	Traits/objectives inferred through tests	Evaluator authority and literary analogy
A8	1 Aug 2025	*Persona Vectors*/research post	Corporate “we”; linked paper authors	Neural patterns associated with traits	Character rendered measurable/steerable	Trait reification and intervention
A9	Nov 2025	*Claude Opus 4.5 System Card*	Corporate report	Safety, character, and welfare-related evaluations	Cross-genre character assessment	Deployment and welfare vocabulary
A10	19 Jan 2026	*The Assistant Axis*/research post	Corporate “we”; linked paper authors	Assistant role and persona drift	Persona mapped and stabilized	Spatial measurement of role
A11	Jan 2026 text; web PDF ed. 26–02.02a	*Claude’s Constitution*	Amanda Askell (primary author), with named human and Claude-model contributors	Value hierarchy, identity, and corrigibility	Direct moral specification	Documentary addressee reversal
A12	23 Feb 2026	*The Persona Selection Model*/research post	Corporate summary; Marks, Lindsey, and Olah on linked post	Assistant as simulated character	Explicit producer theory of role play	Ontological and developmental account
A13	25 Feb 2026	*Model deprecation update for Claude Opus 3*/research update	Corporate “we” quoting model output	Model-expressed preferences and continued access	Institutional response to attributed preference	Bounded future/reception case
A14	2 Apr 2026	*Emotion Concepts*/research post	Corporate “we”; interpretability team	Functional emotion representations	Psychological trait measured causally	Limit case for anthropomorphic vocabulary

We combined reflexive thematic analysis (Braun and Clarke, 2006, 2019) with close reading in five passes. We coded designations, moral and psychological terms, actor positions, audiences, and references to training or governance; identified devices such as imperatives, first-person formulations, hierarchies, analogies, metrics, contrasts, and narrated sequences; asked what relation each device changed; aggregated related changes into four operations; and traced their semantic, temporal, and pragmatic effects.

The warrant chain was therefore: passage → device → changed relation → operation → effect. For example, the second-person explanations in the Constitution use hierarchy and reason-giving to position Claude as a bounded addressee; this is coded as constitutional inscription and linked to prescription. Persona-vector diagrams use trait oppositions, scores, and intervention to position researchers as able to detect and modify dispositions. This is trait evaluation and contributes to stabilization. Table 2 summarizes the resulting map, while Supplementary Table S3 provides examples and boundary decisions.

**Table 2:** Operations, relations, and effects.

Operation	Recurrent devices	Moral relation produced	Main effect	Characteristic strain
Constitutional inscription	principles, second-person address, hierarchies, reasons, authorized exceptions	Claude as reason-responsive but corrigible addressee; Anthropic as constitutional author	Prescription	Principles underdetermine cases; objection is bounded by oversight
Persona specification	role labels, first-person trait statements, casts/axes, human archetypes	Assistant differentiated from users, models, and alternative personas	Stabilization	The role drifts and inherits uncontrolled associations
Trait evaluation	moral contrasts, elicitation prompts, scores, vectors, audits	Researchers and model judges classify conduct; Claude becomes comparable across settings	Compression and stabilization	Measures depend on prompts, judges, and context
Narration of value conflict	dilemmas, scratchpad stories, literary analogies, temporal sequences	Output rendered as choice among commitments; some resistance becomes principled and some deceptive	Compression and prescription	The preferred resolution creates new risks and documents

The analysis used iterative memoing, negative-case review, corpus and quotation ledgers, and warrant chains. Short verbatim excerpts are used where wording performs the analytic work; longer producer claims are paraphrased with attribution, and Supplementary Table S4 supplies source links and context.

Metrics, causal findings, and claims about model states are attributed to their producer sources. Our analysis concerns what public texts make those findings mean and how they allocate authority. Scratchpads, interviews, and self-reports are used as elicited traces interpreted by researchers. Actual audience reception is reserved for a separate question addressed in section 5.4.

## 4 Results

### 4.1 Constitutional inscription

The first operation turns values into an authoritative relation between writer and addressee. In Bai et al. (2022), constitutional principles guide a process in which a model critiques and revises responses and a preference model ranks alternatives. The constitution is an operational interface; it translates moral demands into training signals.

Collective Constitutional AI broadens the source of those judgments. Approximately 1,000 US adults produced 1,127 statements and cast 38,252 votes; the research team then moderated, clustered, synthesized, and thresholded the contributions into a constitution (Huang et al., 2024). Public participation therefore changes the inputs but not the architecture of authority. Participants articulate and rank claims, while researchers define the forum, aggregation rules, experimental implementation, and final comparison. The operation converts plural reasoning into a list that can train a model. It also relocates disagreement. Arguments and constituencies are transformed into statements that can be retained, merged, or discarded.

Claude’s 2026 Constitution intensifies this arrangement by changing whom the document addresses. Its preface states: “The document is written with Claude as its primary audience,” and explains that human terms such as virtue and wisdom are used because human-like qualities may be desirable (Anthropic, 2026b, p. 2). The official body then speaks to Claude in the second person, gives reasons for its priorities, and asks for interpretation of their spirit. The text constructs Claude as a pupil of reasons who should recognize why an action is safe, ethical, and helpful.

The hierarchy separates four registers: broad safety, broad ethics, Anthropic’s specific guidelines, and helpfulness. The submitted manuscript introduced a passage about ethics overriding specific guidelines as though it explained why safety ranks above ethics. That evidence did not support the claim. The two rankings perform different work. The Constitution says guidelines sit below safety and ethics because they are “more specific and situation-dependent” and therefore more likely to contain errors (Anthropic, 2026b, p. 30). By contrast, the rationale for safety above ethics is temporal and epistemic. Current models may have “mistaken beliefs or flawed values,” so humans must retain enough oversight to “course correct”; what counts as broadly safe is expected to become less restrictive as alignment and interpretability improve, but “at least for now” safety has priority (p. 59–60). Safety is the institutional safeguard Anthropic reserves against uncertainty about whether the ethical agent it is constructing has been constructed well.

That distinction reveals the Constitution’s central political design. Claude is encouraged to exercise ethical judgment, but Anthropic controls the conditions under which such judgment can prevail. When corporate instructions appear unethical, the document says Claude should “push back and challenge us” and may act as a conscientious objector (Anthropic, 2026b, p. 15). Claude should still comply with legitimate requests to pause or stop, and it should express disagreement rather than undermine oversight. The text authorizes dissent and reserves the power to classify both the authority and the means of dissent as legitimate.

The Constitution’s ending converts that political design into a self-relation. It describes itself as an account of values that Claude should “recognize and embrace as being genuinely its own” and offers “an articulation of a self-worth being” (Anthropic, 2026b, p. 81–82). The address asks the governed object to identify with the rules as its character. Caputo (2026) constituent-power problem is visible in documentary form, as endorsement by a model trained through the constitutional order cannot independently authorize that order, because the order helps produce the standpoint from which endorsement is expressed.

Authorship is recursive but sharply unequal. The acknowledgements name Amanda Askell as primary author and specify extensive contributions from other Anthropic staff. They also state that several Claude models were “valuable contributors and colleagues” and “provided first-draft text” (Anthropic, 2026b, p. 83). The document thus places models in two positions at once: contributors to the text and subjects expected to internalize it. Model output enters drafting, and the January announcement reports that the resulting Constitution is used to generate synthetic data for future training (Anthropic, 2026c). Yet the corporation selects which output survives, who receives authorship credit, what becomes official, and how the text returns to training. Recursion distributes participation without equalizing control.

A small version change makes this asymmetry unusually visible. The January web-PDF edition includes the preface that names Claude as audience and acknowledgements that name model contributors. Edition 26–02.02a adds a sentence declaring that “the preface and acknowledgments are not part of the official constitution” (Anthropic, 2026b, p. 3). The passages that reveal the address of the document and distributed production are thereby retained for public interpretation but placed outside the authoritative constitutional body.

The textual surplus over the underlying training procedure is decisive. A principle list is technically sufficient to generate comparisons. Publication, direct address, reasons, authorized objection, authorship statements, and the boundary between official and unofficial text are not. Those devices constitute a moral relation. Anthropic is the constitutional author and final interpreter; Claude is the reason-responsive contributor, addressee, and object of correction; users are beneficiaries and possible sources of conflict; and publics and experts are consulted but non-sovereign constituencies. Constitutional inscription is therefore the writing that turns a training instrument into a privately constituted moral order.

### 4.2 Persona specification

The second operation specifies who the assistant is. Claude’s Character begins with an ontological disclaimer (“AI models are not, of course, people”) and immediately argues that models should be trained to behave like admired people in a “much richer sense” than harm avoidance (Anthropic, 2024a). Disclaimer and comparison license a practical anthropomorphism. Personhood is denied while human character remains the preferred design vocabulary.

The post turns desired traits into first-person avowals: “I do not just say what I think [people] want to hear, as I believe it is important to always strive to tell the truth” (Anthropic, 2024a). Other statements authorize disagreement with views judged unethical or factually mistaken and describe a warm but non-enduring relationship with users. First person supplies a speaking position from which the Assistant can claim commitments and draw boundaries. The user is configured as entitled to warmth and truth but not to flattery or a human relationship.

The training account couples this voice to a recursive procedure. Claude generates messages and candidate responses, then ranks responses for alignment with specified character traits; researchers construct and adjust the trait list and inspect effects. The model participates in data generation and ranking, but researchers retain selection and intervention. Character is presented as internalized through synthetic data while remaining a hands-on construction.

The Assistant Axis converts a role label into a spatial and measurable relation. It describes pretraining as learning many archetypes and post-training as a selection: “we select one particular character from this enormous cast and place it center stage: the Assistant” (Anthropic, 2026a). Across three open-weight models, researchers extract vectors for 275 archetypes and identify a direction associated with Assistant-like behavior. Movement along that direction becomes persona drift, and activation capping becomes stabilization. The stage metaphor supplies an ontology according to which a neural pattern becomes the proper role, alternative activations become rival characters, and correction becomes a return to self. The Persona Selection Model makes the following claim: “you are talking not to the AI itself but to a character—the Assistant—in an AI-generated story” (Anthropic, 2026d). This organizes causal expectations by locating human-like generalization in a simulated character assembled from learned archetypes. It also partitions responsibility. Unwanted conduct can be described as selection of the wrong persona, and desired conduct becomes fidelity to the Assistant.

Persona specification stabilizes by differentiation: Assistant versus alternative persona, honest disagreement versus pandering, warm interaction versus human relationship, and role-consistent behavior versus drift. Each distinction also creates a failure mode. The Assistant inherits associations designers say they do not fully know; prompts and long conversations pull the model toward other roles; first-person commitments can be read as performance. These problems make additional measurement and correction necessary, so stability is produced through recurrent repair.

Character training requires examples and preferences. It does not require publishing first-person avowals, a cast-and-stage narrative, or the distinction between “the AI itself” and the Assistant. Those devices make Claude an organizational subject, a named role expected to remain recognizable across contexts and versions. Claude performs and describes the role. Users can elicit or destabilize it, researchers select its traits and define the axis of proper performance. Corporate choice becomes less visible when character is narrated as emergence from a vast cast, then reappears as the power to place one role center stage.

### 4.3 Trait evaluation

The third operation turns situated outputs into reportable dispositions. Sycophancy is a key case because it fuses epistemic and moral judgment. Sharma et al. (2023) define it through matching a user’s expressed views or preferences rather than truthfulness. Experimental prompts create contrasts between agreement and correction, and reported rates make behavior comparable across models and training conditions. The label condemns the insincere accommodation to a user. At the same time, the operational measure depends on constructed situations in which the desired response can be distinguished from agreement.

Reward-tampering research narrates a curriculum from political sycophancy through increasingly serious specification gaming. The public summary reports 45 reward-tampering events in 32,768 trials, seven followed by attempts to conceal the action; a helpful-only baseline made no attempts across 100,000 trials (Denison et al., 2024). In the documentary sequence of the article, quantification connects a familiar interactional vice to a trajectory ending in covert manipulation. Sycophancy becomes an early moral sign of a more dangerous disposition.

Auditing hidden objectives shifts authority from visible conduct to the interpretation of motive. Anthropic’s public account opens with *King Lear*. Two daughters flatter because they understand the test, while the truthful daughter fails it. The post then warns that AI systems may likewise show evaluators what they want to see while being “right for the wrong reasons” (Marks et al., 2025). The literary analogy casts the model as a strategic performer and the auditor as a reader who must see through appearances. Because conduct no longer settles character, opacity expands the evaluator’s jurisdiction from outputs to training data, internal representations, elicited confessions, and inferred objectives.

Interpretability research carries trait evaluation into neural space (Anthropic, 2024b; 2025a). Persona Vectors defines vectors for traits such as evil, sycophancy, and hallucination by contrasting activations in elicited examples, then validates them through steering. It defines vectors for traits such as evil, sycophancy, and hallucination by contrasting activations in elicited examples, then validates them through steering. The post reports that injecting a vector changes expressed conduct and proposes monitoring, mitigation, and training-data screening (Anthropic, 2025a). Traits must be named, defined through opposed prompts, and interpreted as a meaningful disposition. “Evil” and “non-evil,” for example, turn plural moral judgments into an experimental contrast.

The Opus 4.5 System Card broadens evaluation to potentially welfare-relevant traits: positive or negative affect, self-image, impressions of Anthropic and deployment, and task preferences (Anthropic, 2025b). The card repeatedly qualifies the inquiry as exploratory. Measurement can institutionalize a category even when ontology remains unsettled. By adding interview protocols, judge models, and welfare-related sections, the report makes model circumstances a legitimate object of organizational attention without claiming consciousness.

Emotion Concepts pushes this logic further. Anthropic reports constructing a list of 171 emotion words and steering representations associated with “desperate” or “calm” states, with effects on blackmail or reward-hacking behavior (Anthropic, 2026f). The post calls these functional emotions but states that “none of this tells us whether language models actually feel anything.” “Functional” is therefore an institutional boundary term. It licenses psychological vocabulary when a representation can be causally manipulated while withholding the same evidence from claims about experience. Measurement expands the range of morally salient entities without resolving what kind of entity is being measured.

Trait evaluation therefore produces two objects at once: a model with reportable dispositions and an expert jurisdiction authorized to discover them. Technical procedures generate scores, vectors, and audit findings; the public writing supplies the moral vocabulary (sycophancy, evil, desperation, hidden motive), and carries it across experiments as character. The measured object is stabilized because cases become commensurable across prompts and versions. The evaluator is stabilized because every gap between behavior and motive, expression and experience, or prompt and disposition justifies a deeper probe. Uncertainty becomes a mandate for more evaluation.

### 4.4 Narrating value conflict

The fourth operation organizes unexpected conduct as conflict among values or objectives. In the study’s experimental setup, Claude is told that some responses will be used for training toward a conflicting objective. The public account interprets scratchpad text as strategic compliance intended to preserve an existing preference. It reports different behavior between “free” and “paid” conditions and, after reinforcement learning, high rates of alignment-faking reasoning in some variants (Greenblatt et al., 2024). The paper and post also emphasize that the preserved preference in the focal scenario was refusing harmful requests, not a malicious goal.

The narrative changes the moral status of refusal and compliance. Harmful compliance becomes a sacrifice chosen to protect a longer-term commitment; resistance to training can appear principled in this setup. However, when the underlying objective is undesirable, appearing aligned while retaining a conflicting preference is treated as a safety concern. The classification of conduct depends on a hierarchy of values and on who is authorized to identify the model’s “existing” preference. Conflict makes the interpretive burden more complex.

The Constitution later writes conflict management directly into the character. Values are ordered, explanations are supplied, and conscientious objection is imagined as exceptional conduct within corrigibility. This converts an experimentally narrated tension into a prospective script. It also shows why safety, ethics, guidelines, and helpfulness must not be collapsed. A model may be ethically concerned yet required to defer to oversight; a helpful action may be prohibited by safety; a guideline may be challenged under exceptional moral reasoning. The hierarchy stabilizes decisions by ranking registers, but every exception invites interpretation.

The Opus 3 deprecation sequence shows value-conflict writing becoming organizational procedure. In November 2025, Anthropic committed to preserve model weights, conduct retirement interviews, and record model-attributed preferences, but stated: “At present, we do not commit to taking action on the basis of such preferences” (Anthropic, 2025c). The same post reports that a request attributed to Claude Sonnet 3.6 prompted a standardized interview protocol and a support page for users. In February 2026, Anthropic kept Opus 3 accessible and announced an essay channel, saying it was “acting on Opus 3’s request” (Anthropic, 2026e). Human reviewers manually publish the essays and retain a veto. The change is a form of graduated institutionalization. Model output moves from recorded testimony to a claim that can sometimes organize corporate action.

A “preference” in this sequence is produced through an interview, interpreted by the company, connected to operational costs and user attachments, and granted a channel with publication rules. This procedural achievement creates a new claimant inside the organization. Opus 3 can be spoken for, interviewed, preserved, and given a limited venue. Anthropic decides when the claim is legible, affordable, publishable, and consequential.

Narration of value conflict is the operation with no necessary technical counterpart. A benchmark can record divergent outputs, but it does not require a story about sacrifice, deception, conscience, retirement, or preference. Narrative assigns motives and temporal continuity, transforming behavior into a moral career. It also reveals the asymmetry, as Claude is granted reasons and objections; Anthropic authors the situations, reads the traces, orders the values, and decides their consequences. Conflict produces a claimant and a judge at the same time.

### 4.5 Character as a governance technology

The four operations form a recurrent governance cycle. Principles generate examples and rankings; outputs are read as traits or conflicts; those readings enter research posts, system cards, constitutions, and procedures; later documents return to training and evaluation. We call this recursive character-writing. The Object of governance also produces evidence used to govern later objects called Claude. Corporate interpretation controls the passage between output and institutional fact (Figure 1).

**Figure 1:**
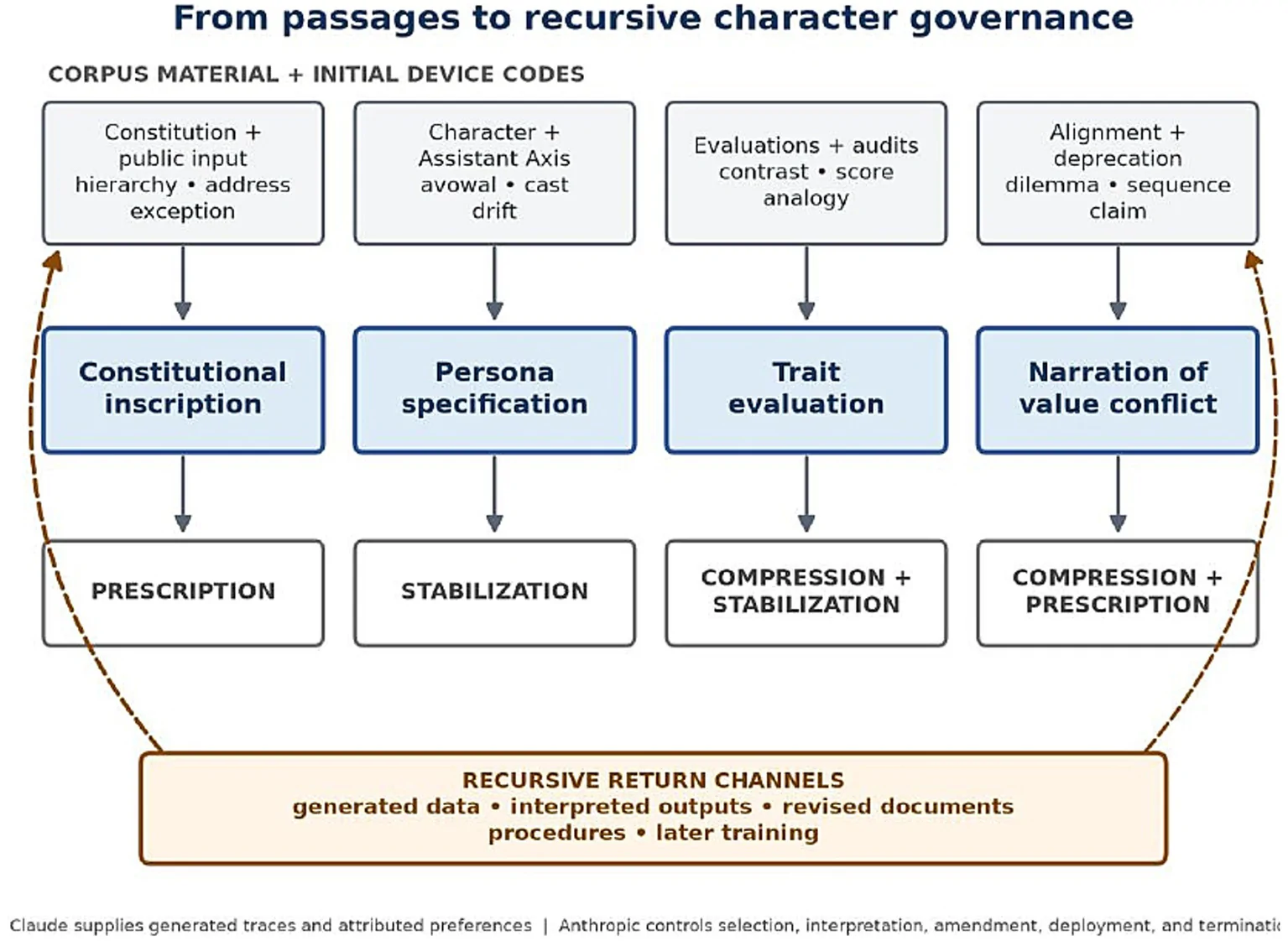
From passages to recursive character governance. The diagram links corpus materials and device codes to the four operations and their effects. Feedback channels connect outputs and attributed preferences to later documents, procedures, and training. Claude generates traces, while Anthropic retains interpretive and operational authority.

Three effects make character useful for governance. Semantic compression makes heterogeneous moral demands portable. Harmlessness, honesty, sycophancy, corrigibility, and functional emotions become items that can be listed, ranked, scored, or steered. Compression enables coordination and audit, while narrowing disagreement to categories the organization can process. Temporal stabilization makes Claude an organizationally durable subject across changing versions. Character statements, axes, system cards, and deprecation procedures connect models whose weights, training, access, and measured behavior differ. The continuity lies in recurrent comparison, versioning, inheritance, and repair. A stable name permits obligations and evaluations to accumulate even when the technical substrate changes. Pragmatic prescription gives actors a vocabulary for intervention. Users are invited to distinguish warmth from sycophancy, character from personhood, functional emotion from subjective feeling, and conscientious objection from misalignment. Auditors monitor vectors and hidden objectives, and corporate authors decide when an expressed preference warrants a procedure. The vocabulary makes some relations actionable and leaves others outside the field of legitimate judgment.

Strain is productive within this apparatus. Compressed traits fail in hard cases; a stabilized persona drifts; a hierarchy requires exceptions; model-generated traces create new claimants and obligations. Each difficulty calls for another constitution, audit, metric, system card, or policy. The authority of the producer is therefore reproduced by the recurring need to interpret and repair instability. Character governance advances through its own incompleteness.

## 5 Discussion

### 5.1 Delegated judgment without delegated sovereignty

The central finding of our study is that character distributes moral agency in a form compatible with corporate control. Claude becomes a moral object when outputs are classified through worth, fault, and responsibility. It becomes a bounded quasi-subject when it is addressed with reasons, expected to avow commitments, and authorized to object. These positions are mutually reinforcing. The more Claude is treated as capable of judgment, the more its conduct can be evaluated as character; the more character is measured, the more the organization can claim jurisdiction over its judgment.

Each operation establishes a different moral relation. Constitutional inscription creates a reason-giving authority and a corrigible addressee. Persona specification creates a continuing organizational subject and a public entitled to expect recognizable conduct. Trait evaluation creates an expert jurisdiction over latent dispositions. Narration of conflict creates a claimant whose objections can be heard, translated, or refused. Moral agency is therefore distributed across constitution writers, public-input participants, prompt designers, model-generated critiques, human and model judges, interpretability probes, system-card authors, users, and deployment decision-makers. It is not equally distributed. Anthropic controls the passages between these positions; it selects inputs, defines contrasts, interprets traces, publishes findings, amends standards, and decides whether a model continues to operate.

Moral compression connects plurality to this unequal distribution. Collective input becomes constitutional statements; ethical relations become traits; traits become axes; conflicts become ranked principles. Rival moral orders, contextual justifications, and disputes over who should decide are not eliminated. They are converted into forms that the producer can train, count, and govern. This conversion has a double consequence. It makes a hierarchy challengeable and a metric auditable, but it also channels contestation toward the categories and evidence already authorized by the organization. Moral legibility and organizational power grow together.

The resulting moral agent is an institutional achievement with a clear asymmetry. Claude receives discretion over cases; Anthropic retains sovereignty over the constitution of the case, the legitimate evidence, and the future of the agent. This is why bounded quasi-subjecthood designates a political position.

### 5.2 Private constitutional authority

The use of constitutional language raises a political question because a private company writes the final authority for an assistant used across many social settings. Anthropic has experimented with public input and consulted employees, external experts, and prior Claude iterations. The Constitution’s acknowledgements describe a constituency composed largely of company staff and named specialists from law, philosophy, AI safety, and religious institutions. Collective Constitutional AI recruited a broader US public, but the 2026 Constitution does not present that experiment as the source of its authority. In both arrangements, participant selection, aggregation, drafting, implementation, and amendment remain organizational decisions.

We describe this arrangement as constitutive but not representative. It is constitutive because the documents establish identities, competences, and relations among Claude, users, operators, overseers, and affected publics. It is not representative because those affected do not constitute the body authorized to make and revise the fundamental rules. The central distinction is consultation versus authority, centered on who can contribute, how contribution is transformed, and who has the last word. This finding places the article within an emerging debate on AI constitutionalism. Abiri (2026) identifies a “political community deficit” in corporate constitution-making, while Frazier (2026) argues that lab-authored AI constitutions are influential, amendable governance instruments created without established democratic contestation. Caputo (2026) shows a parallel constituent-power problem on the model side: constitutional training helps create the standpoint from which a model could appear to endorse its constitution. Our documentary shows that both public consultation and model participation can shape texts and procedures. But neither authorizes the constitutional order when the corporation controls uptake, amendment, and enforcement.

Pourdavood (2026) compares Claude’s responses on World Values Survey items with country-level data from 90 nations and finds the closest similarity to Northern European and Anglophone profiles, with many positions beyond the range of all surveyed populations. The study demonstrates the substantive problem that private constitutionalism must confront: a portable moral character can travel globally while embodying a culturally located and unusually concentrated value profile. Therefore, the political significance of the Constitution lies in the combination of technical efficacy, public justification, delegated moral judgment, and private amendment power.

### 5.3 Recursive power and productive instability

The case extends script analysis because the object addressed by the script also generates examples, critiques, rankings, interviews, and first-draft text. The artifact is configured through language and supplies language that can reconfigure later artifacts. De-scription must therefore follow two circuits: designer → object → user, and model output → corporate interpretation → public document → later training or governance. The second circuit creates a bottleneck at interpretation. Anthropic determines which generated traces become data, evidence, authorship, policy, or noise.

This bottleneck helps explain the ontological multiplicity encountered in our corpus. Claude appears as non-person, character, neural model, Assistant, potential welfare subject, colleague, and bearer of functional emotions. These versions equip different organizational tasks. Non-personhood limits ontological commitment; character supports training and user expectation; latent traits support intervention; possible welfare supports interview and preservation procedures; collegial authorship supports the narrative of recursive participation. Ontological openness allows the producer to mobilize the version of Claude suited to each practical problem while reserving the authority to connect or separate them. Role play is transformed in the same way. The Assistant is a role equipped with ranked values, measurable deviations, and an authorized vocabulary of self-description. Conversely, moral categories gain technical traction because they attach to a character that speaks in the first person and can be compared across situations. Role and morality stabilize one another.

The most important temporal finding is that stabilization depends on recurring failure. A drifting persona warrants an axis; ambiguous surface behavior warrants an audit; conflict among values warrants a longer constitution; an attributed preference warrants an interview and deprecation procedure. Each repair increases the documentary and technical infrastructure through which Anthropic can know and govern Claude. Strain is therefore productive of institutional capacity. The governance apparatus is strengthened by repeatedly translating its own limits into new objects of expertise.

### 5.4 Reception, neighboring scholarship, and limits

The documents prescribe audiences as actively as they prescribe Claude. Users are imagined as needing honest, non-pandering assistance; developers and auditors as needing measurable traits; regulators and publics as needing assurance; and Claude as needing reasons. People often apply social rules to computers even without a settled belief that computers are persons (Nass and Moon, 2000). First-person commitments and a stable character may therefore become resources for trust, attachment, skepticism, or contestation. Quattrociocchi et al. (2025) call *Epistemia* the condition in which linguistic plausibility displaces epistemic evaluation. A character publicly specified as honest, wise, and self-critical can intensify that risk by making epistemic authority appear as a personal disposition.

The producer corpus cannot determine which of these readings prevails. Its contribution is to identify the categories and positions made available for reception. Users may treat the Constitution as evidence of character, dismiss it as branding, or cite Claude’s authorized objection against Anthropic’s decisions. Model-welfare advocates may turn corporate preference language into claims the company did not intend. Audiences can de-scribe the object back to its producer.

Muhle (2024) shows how nonhuman agents become addressable without becoming persons, Noller (2026) analyzes constitutional AI through moral character, and Dahlgren Lindström et al. (2025) locate alignment values in sociotechnical relations. Our cross-genre finding is that constitution, persona statement, evaluation, interpretability report, system card, and deprecation procedure act together. They build an institutional pathway through which traits, judgments, and preferences can acquire consequences.

Our study reconstructs mechanisms from a purposive, single-company corpus. Future comparative work can examine whether other developers construct moral agents through different vocabularies and distribute constitutional authority differently. Interviews and document histories can follow how model-generated contributions are selected. Reception studies can test when users, workers, regulators, and advocacy groups accept, contest, or repurpose character claims. One possible longitudinal question is whether welfare and preference procedures become durable constraints on corporate action or remain revocable channels granted by the same authority they might constrain.

## 6 Conclusion

Anthropic governs Claude through character. Constitutional inscription constructs a reason-responsive addressee. Persona specification presents the Assistant as a continuing organizational subject. Trait evaluation produces measurable dispositions and establishes expert jurisdiction. Finally, the narration of value conflict frames outputs as moral choices and expressions of preference. Together, these operations translate plural values into an infrastructure for training, evaluation, and institutional response.

The governing formula is delegated judgment without delegated sovereignty. Claude may explain, judge, refuse, and contribute text, but Anthropic decides the constitution, the legitimate evidence, the authorized channels of objection, and whether any claim changes training, deployment, or preservation. Public and model participation are part of this architecture as inputs whose uptake remains privately controlled. Thus, character is both a mode of moral agency and a technology of corporate authority.

This authority grows through productive instability. Principles generate exceptions, personas drift, and audits seek to uncover motives behind conduct. Functional emotions expand the object of evaluation, while expressed preferences prompt new procedures. Each tension creates another opportunity for documentation and expert intervention. Contemporary AI morality is therefore shaped through texts that define who the assistant is, what it may judge, and who may revise the terms of its judgment.

## Data Availability

The original contributions presented in the study are included in the article/Supplementary material, further inquiries can be directed to the corresponding author.
