# Supplementary material for: Governing AI through character: moral agency and corporate authority in Anthropic’s public texts
Source: Front Sociol. 2026 Sep 14;11:1912778. doi: 10.3389/fsoc.2026.1912778 (PMC13616688; doi:10.3389/fsoc.2026.1912778)
Supplement: Supplementary file 1 [file Table_1.DOCX]

**Supplementary material**

# Table S1. 130-item producer-side frame

| **ID** | **Displayed date / version** | **Title** | **Channel / genre** | **Inclusion status** | **URL** |
| --- | --- | --- | --- | --- | --- |
| 1 | December 1, 2021 | A General Language Assistant as a Laboratory for Alignment. | Research page / linked publication | Frame only | https://www.anthropic.com/research/a-general-language-assistant-as-a-laboratory-for-alignment |
| 2 | April 12, 2022 | Training a Helpful and Harmless Assistant with Reinforcement Learning from Human Feedback. | Research page / linked publication | Frame only | https://www.anthropic.com/research/training-a-helpful-and-harmless-assistant-with-reinforcement-learning-from-human |
| 3 | July 11, 2022 | Language Models (Mostly) Know What They Know. | Research page / linked publication | Frame only | https://www.anthropic.com/research/language-models-mostly-know-what-they-know |
| 4 | November 4, 2022 | Measuring Progress on Scalable Oversight for Large Language Models. | Research page / linked publication | Frame only | https://www.anthropic.com/research/measuring-progress-on-scalable-oversight-for-large-language-models |
| 5 | December 15, 2022 | Constitutional AI: Harmlessness from AI Feedback. | Research page / linked publication | Analytic subcorpus | https://www.anthropic.com/research/constitutional-ai-harmlessness-from-ai-feedback |
| 6 | December 19, 2022 | Discovering Language Model Behaviors with Model-Written Evaluations. | Research page / linked publication | Frame only | https://www.anthropic.com/research/discovering-language-model-behaviors-with-model-written-evaluations |
| 7 | December 19, 2022 | Tracing Model Outputs to the Training Data. | Research page / linked publication | Frame only | https://www.anthropic.com/research/influence-functions |
| 8 | July 18, 2023 | Measuring Faithfulness in Chain-of-Thought Reasoning. | Research page / linked publication | Frame only | https://www.anthropic.com/research/measuring-faithfulness-in-chain-of-thought-reasoning |
| 9 | July 18, 2023 | Question Decomposition Improves the Faithfulness of Model-Generated Reasoning. | Research page / linked publication | Frame only | https://www.anthropic.com/research/question-decomposition-improves-the-faithfulness-of-model-generated-reasoning |
| 10 | August 8, 2023 | Studying Large Language Model Generalization with Influence Functions. | Research page / linked publication | Frame only | https://www.anthropic.com/research/studying-large-language-model-generalization-with-influence-functions |
| 11 | October 23, 2023 | Towards Understanding Sycophancy in Language Models. | Research page / linked publication | Analytic subcorpus | https://www.anthropic.com/research/towards-understanding-sycophancy-in-language-models |
| 12 | October 24, 2023 | Specific versus General Principles for Constitutional AI. | Research page / linked publication | Frame only | https://www.anthropic.com/research/specific-versus-general-principles-for-constitutional-ai |
| 13 | January 14, 2024 | Sleeper Agents: Training Deceptive LLMs that Persist Through Safety Training. | Research page / linked publication | Frame only | https://www.anthropic.com/research/sleeper-agents-training-deceptive-llms-that-persist-through-safety-training |
| 14 | April 2, 2024 | Many-shot jailbreaking. | Research page / linked publication | Frame only | https://www.anthropic.com/research/many-shot-jailbreaking |
| 15 | April 23, 2024 | Simple probes can catch sleeper agents. | Research page / linked publication | Frame only | https://www.anthropic.com/research/probes-catch-sleeper-agents |
| 16 | June 8, 2024 | Claude’s Character. | Research page / linked publication | Analytic subcorpus | https://www.anthropic.com/research/claude-character |
| 17 | June 17, 2024 | Sycophancy to subterfuge: Investigating reward tampering in language models. | Research page / linked publication | Analytic subcorpus | https://www.anthropic.com/research/reward-tampering |
| 18 | October 18, 2024 | Sabotage evaluations for frontier models. | Research page / linked publication | Frame only | https://www.anthropic.com/research/sabotage-evaluations |
| 19 | December 18, 2024 | Alignment faking in large language models. | Research page / linked publication | Analytic subcorpus | https://www.anthropic.com/research/alignment-faking |
| 20 | February 3, 2025 | Constitutional Classifiers: Defending against universal jailbreaks. | Research page / linked publication | Frame only | https://www.anthropic.com/research/constitutional-classifiers |
| 21 | February 25, 2025 | Forecasting rare language model behaviors. | Research page / linked publication | Frame only | https://www.anthropic.com/research/forecasting-rare-behaviors |
| 22 | March 13, 2025 | Auditing language models for hidden objectives. | Research page / linked publication | Analytic subcorpus | https://www.anthropic.com/research/auditing-hidden-objectives |
| 23 | April 3, 2025 | Reasoning models don’t always say what they think. | Research page / linked publication | Frame only | https://www.anthropic.com/research/reasoning-models-dont-say-think |
| 24 | April 24, 2025 | Exploring model welfare. | Research page / linked publication | Frame only | https://www.anthropic.com/research/exploring-model-welfare |
| 25 | June 16, 2025 | SHADE-Arena: Evaluating Sabotage and Monitoring in LLM Agents. | Research page / linked publication | Frame only | https://www.anthropic.com/research/shade-arena-sabotage-monitoring |
| 26 | June 20, 2025 | Agentic Misalignment: How LLMs could be insider threats. | Research page / linked publication | Frame only | https://www.anthropic.com/research/agentic-misalignment |
| 27 | August 15, 2025 | Claude Opus 4 and 4.1 can now end a rare subset of conversations. | Research page / linked publication | Frame only | https://www.anthropic.com/research/end-subset-conversations |
| 28 | October 6, 2025 | Petri: An open-source auditing tool to accelerate AI safety research. | Research page / linked publication | Frame only | https://www.anthropic.com/research/petri-open-source-auditing |
| 29 | October 9, 2025 | A small number of samples can poison LLMs of any size. | Research page / linked publication | Frame only | https://www.anthropic.com/research/small-samples-poison |
| 30 | November 4, 2025 | Commitments on model deprecation and preservation. | Research page / linked publication | Revision-stage comparison | https://www.anthropic.com/research/deprecation-commitments |
| 31 | November 21, 2025 | From shortcuts to sabotage: natural emergent misalignment from reward hacking. | Research page / linked publication | Frame only | https://www.anthropic.com/research/emergent-misalignment-reward-hacking |
| 32 | December 19, 2025 | Introducing Bloom: an open source tool for automated behavioral evaluations. | Research page / linked publication | Frame only | https://www.anthropic.com/research/bloom |
| 33 | January 9, 2026 | Next-generation Constitutional Classifiers: More efficient protection against universal jailbreaks. | Research page / linked publication | Frame only | https://www.anthropic.com/research/next-generation-constitutional-classifiers |
| 34 | January 28, 2026 | Disempowerment patterns in real-world AI usage. | Research page / linked publication | Frame only | https://www.anthropic.com/research/disempowerment-patterns |
| 35 | January 29, 2026 | How AI assistance impacts the formation of coding skills. | Research page / linked publication | Frame only | https://www.anthropic.com/research/AI-assistance-coding-skills |
| 36 | February 23, 2026 | The Persona Selection Model. | Research page / linked publication | Analytic subcorpus | https://www.anthropic.com/research/persona-selection-model |
| 37 | February 25, 2026 | An update on our model deprecation commitments for Claude Opus 3. | Research page / linked publication | Analytic subcorpus | https://www.anthropic.com/research/deprecation-updates-opus-3 |
| 38 | December 22, 2021 | A Mathematical Framework for Transformer Circuits. | Research page / linked publication | Frame only | https://www.anthropic.com/research/a-mathematical-framework-for-transformer-circuits |
| 39 | March 8, 2022 | In-context Learning and Induction Heads. | Research page / linked publication | Frame only | https://www.anthropic.com/research/in-context-learning-and-induction-heads |
| 40 | May 21, 2022 | Scaling Laws and Interpretability of Learning from Repeated Data. | Research page / linked publication | Frame only | https://www.anthropic.com/research/scaling-laws-and-interpretability-of-learning-from-repeated-data |
| 41 | June 17, 2022 | Softmax Linear Units. | Research page / linked publication | Frame only | https://www.anthropic.com/research/softmax-linear-units |
| 42 | September 14, 2022 | Toy Models of Superposition. | Research page / linked publication | Frame only | https://www.anthropic.com/research/toy-models-of-superposition |
| 43 | January 5, 2023 | Superposition, Memorization, and Double Descent. | Research page / linked publication | Frame only | https://www.anthropic.com/research/superposition-memorization-and-double-descent |
| 44 | March 16, 2023 | Privileged Bases in the Transformer Residual Stream. | Research page / linked publication | Frame only | https://www.anthropic.com/research/privileged-bases-in-the-transformer-residual-stream |
| 45 | May 4, 2023 | Distributed Representations: Composition & Superposition. | Research page / linked publication | Frame only | https://www.anthropic.com/research/distributed-representations-composition-superposition |
| 46 | May 24, 2023 | Circuits Updates — May 2023. | Research page / linked publication | Frame only | https://www.anthropic.com/research/circuits-updates-may-2023 |
| 47 | May 24, 2023 | Interpretability Dreams. | Research page / linked publication | Frame only | https://www.anthropic.com/research/interpretability-dreams |
| 48 | October 5, 2023 | Decomposing Language Models Into Understandable Components. | Research page / linked publication | Frame only | https://www.anthropic.com/research/decomposing-language-models-into-understandable-components |
| 49 | October 5, 2023 | Towards Monosemanticity: Decomposing Language Models With Dictionary Learning. | Research page / linked publication | Frame only | https://www.anthropic.com/research/towards-monosemanticity-decomposing-language-models-with-dictionary-learning |
| 50 | March 8, 2024 | Reflections on Qualitative Research. | Research page / linked publication | Frame only | https://www.anthropic.com/research/transformer-circuits |
| 51 | April 26, 2024 | Circuits Updates – April 2024. | Research page / linked publication | Frame only | https://www.anthropic.com/research/circuits-updates-april-2024 |
| 52 | May 21, 2024 | Mapping the Mind of a Large Language Model. | Research page / linked publication | Frame only | https://www.anthropic.com/research/mapping-mind-language-model |
| 53 | June 13, 2024 | The engineering challenges of scaling interpretability. | Research page / linked publication | Frame only | https://www.anthropic.com/research/engineering-challenges-interpretability |
| 54 | June 28, 2024 | Circuits Updates – June 2024. | Research page / linked publication | Frame only | https://www.anthropic.com/research/circuits-updates-june-2024 |
| 55 | July 31, 2024 | Circuits Updates – July 2024. | Research page / linked publication | Frame only | https://www.anthropic.com/research/circuits-updates-july-2024 |
| 56 | September 6, 2024 | Circuits Updates – August 2024. | Research page / linked publication | Frame only | https://www.anthropic.com/research/circuits-updates-august-2024 |
| 57 | October 1, 2024 | Circuits Updates – September 2024. | Research page / linked publication | Frame only | https://www.anthropic.com/research/circuits-updates-sept-2024 |
| 58 | October 16, 2024 | Using dictionary learning features as classifiers. | Research page / linked publication | Frame only | https://www.anthropic.com/research/features-as-classifiers |
| 59 | October 25, 2024 | Evaluating feature steering: A case study in mitigating social biases. | Research page / linked publication | Frame only | https://www.anthropic.com/research/evaluating-feature-steering |
| 60 | February 20, 2025 | Insights on Crosscoder Model Diffing. | Research page / linked publication | Frame only | https://www.anthropic.com/research/crosscoder-model-diffing |
| 61 | March 27, 2025 | Tracing the thoughts of a large language model. | Research page / linked publication | Frame only | https://www.anthropic.com/research/tracing-thoughts-language-model |
| 62 | May 29, 2025 | Open-sourcing circuit-tracing tools. | Research page / linked publication | Frame only | https://www.anthropic.com/research/open-source-circuit-tracing |
| 63 | August 1, 2025 | Persona vectors: Monitoring and controlling character traits in language models. | Research page / linked publication | Analytic subcorpus | https://www.anthropic.com/research/persona-vectors |
| 64 | October 29, 2025 | Emergent introspective awareness in large language models. | Research page / linked publication | Frame only | https://www.anthropic.com/research/introspection |
| 65 | January 19, 2026 | The assistant axis: situating and stabilizing the character of large language models. | Research page / linked publication | Analytic subcorpus | https://www.anthropic.com/research/assistant-axis |
| 66 | March 13, 2026 | A”diff" tool for AI: Finding behavioral differences in new models. | Research page / linked publication | Frame only | https://www.anthropic.com/research/diff-tool |
| 67 | April 2, 2026 | Emotion concepts and their function in a large language model. | Research page / linked publication | Analytic subcorpus | https://www.anthropic.com/research/emotion-concepts-function |
| 68 | February 15, 2022 | Predictability and Surprise in Large Generative Models. | Research page / linked publication | Frame only | https://www.anthropic.com/research/predictability-and-surprise-in-large-generative-models |
| 69 | August 22, 2022 | Red Teaming Language Models to Reduce Harms: Methods, Scaling Behaviors, and Lessons Learned. | Research page / linked publication | Frame only | https://www.anthropic.com/research/red-teaming-language-models-to-reduce-harms-methods-scaling-behaviors-and-lessons-learned |
| 70 | February 15, 2023 | The Capacity for Moral Self-Correction in Large Language Models. | Research page / linked publication | Frame only | https://www.anthropic.com/research/the-capacity-for-moral-self-correction-in-large-language-models |
| 71 | October 17, 2023 (public research page; conference paper 2024) | Collective Constitutional AI: Aligning a Language Model with Public Input. | Research page / linked publication | Analytic subcorpus | https://www.anthropic.com/research/collective-constitutional-ai-aligning-a-language-model-with-public-input |
| 72 | June 29, 2023 | Towards Measuring the Representation of Subjective Global Opinions in Language Models. | Research page / linked publication | Frame only | https://www.anthropic.com/research/towards-measuring-the-representation-of-subjective-global-opinions-in-language-models |
| 73 | December 7, 2023 | Evaluating and Mitigating Discrimination in Language Model Decisions. | Research page / linked publication | Frame only | https://www.anthropic.com/research/evaluating-and-mitigating-discrimination-in-language-model-decisions |
| 74 | April 9, 2024 | Measuring the Persuasiveness of Language Models. | Research page / linked publication | Frame only | https://www.anthropic.com/research/measuring-model-persuasiveness |
| 75 | June 6, 2024 | Testing and mitigating elections-related risks. | Research page / linked publication | Frame only | https://www.anthropic.com/research/testing-and-mitigating-elections-related-risks |
| 76 | December 12, 2024 | Clio: Privacy-preserving insights into real-world AI use. | Research page / linked publication | Frame only | https://www.anthropic.com/research/clio |
| 77 | February 10, 2025 | Introducing the Anthropic Economic Index. | News or policy announcement | Frame only | https://www.anthropic.com/news/the-anthropic-economic-index |
| 78 | March 13, 2025 | What 81,000 Conversations Tell Us about Work with AI. | News or policy announcement | Frame only | https://www.anthropic.com/news/81k-interviews |
| 79 | March 27, 2025 | Anthropic Economic Index: Insights from Claude 3.7 Sonnet. | News or policy announcement | Frame only | https://www.anthropic.com/news/anthropic-economic-index-insights-from-claude-sonnet-3-7 |
| 80 | April 8, 2025 | Anthropic Education Report: How University Students Use Claude. | News or policy announcement | Frame only | https://www.anthropic.com/news/anthropic-education-report-how-university-students-use-claude |
| 81 | April 21, 2025 | Values in the wild: Discovering and analyzing values in real-world language model interactions. | Research page / linked publication | Frame only | https://www.anthropic.com/research/values-wild |
| 82 | April 28, 2025 | Anthropic Economic Index: AI’s impact on software development. | Research page / linked publication | Frame only | https://www.anthropic.com/research/impact-software-development |
| 83 | June 27, 2025 | How people use Claude for support, advice, and companionship. | News or policy announcement | Frame only | https://www.anthropic.com/news/how-people-use-claude-for-support-advice-and-companionship |
| 84 | August 27, 2025 | Anthropic education report: How educators use Claude. | News or policy announcement | Frame only | https://www.anthropic.com/news/anthropic-education-report-how-educators-use-claude |
| 85 | December 2, 2025 | How AI Is Transforming Work at Anthropic. | Research page / linked publication | Frame only | https://www.anthropic.com/research/how-ai-is-transforming-work-at-anthropic |
| 86 | December 4, 2025 | Introducing Anthropic Interviewer. | Research page / linked publication | Frame only | https://www.anthropic.com/research/anthropic-interviewer |
| 87 | February 18, 2026 | Measuring AI agent autonomy in practice. | Research page / linked publication | Frame only | https://www.anthropic.com/research/measuring-agent-autonomy |
| 88 | September 19, 2023 | Challenges in evaluating AI systems. | Research page / linked publication | Frame only | https://www.anthropic.com/research/evaluating-ai-systems |
| 89 | November 19, 2024 | A statistical approach to model evaluations. | Research page / linked publication | Frame only | https://www.anthropic.com/research/statistical-approach-to-model-evals |
| 90 | December 19, 2024 | Building Effective AI Agents. | Research page / linked publication | Frame only | https://www.anthropic.com/research/building-effective-agents |
| 91 | January 6, 2025 | Claude SWE-Bench Performance. | Research page / linked publication | Frame only | https://www.anthropic.com/research/swe-bench-sonnet |
| 92 | February 24, 2025 | Claude’s extended thinking. | Research page / linked publication | Frame only | https://www.anthropic.com/research/visible-extended-thinking |
| 93 | June 18, 2025 | Confidential Inference via Trusted Virtual Machines. | Research page / linked publication | Frame only | https://www.anthropic.com/research/confidential-inference-trusted-vms |
| 94 | June 27, 2025 | Project Vend: Can Claude run a small shop? (And why does that matter?). | Research page / linked publication | Frame only | https://www.anthropic.com/research/project-vend-1 |
| 95 | September 15, 2025 | Anthropic Economic Index report: Uneven geographic and enterprise AI adoption. | Research page / linked publication | Frame only | https://www.anthropic.com/research/anthropic-economic-index-september-2025-report |
| 96 | September 15, 2025 | Anthropic Economic Index: Tracking AI’s role in the US and global economy. | Research page / linked publication | Frame only | https://www.anthropic.com/research/economic-index-geography |
| 97 | October 3, 2025 | Building AI for cyber defenders. | Research page / linked publication | Frame only | https://www.anthropic.com/research/building-ai-cyber-defenders |
| 98 | October 14, 2025 | Preparing for AI’s economic impact: exploring policy responses. | Research page / linked publication | Frame only | https://www.anthropic.com/research/economic-policy-responses |
| 99 | November 12, 2025 | Project Fetch: Can Claude train a robot dog?. | Research page / linked publication | Frame only | https://www.anthropic.com/research/project-fetch-robot-dog |
| 100 | November 24, 2025 | Mitigating the risk of prompt injections in browser use. | Research page / linked publication | Frame only | https://www.anthropic.com/research/prompt-injection-defenses |
| 101 | November 25, 2025 | Estimating AI productivity gains. | Research page / linked publication | Frame only | https://www.anthropic.com/research/estimating-productivity-gains |
| 102 | December 18, 2025 | Project Vend: Phase two. | Research page / linked publication | Frame only | https://www.anthropic.com/research/project-vend-2 |
| 103 | January 15, 2026 | Anthropic Economic Index report: Economic primitives. | Research page / linked publication | Frame only | https://www.anthropic.com/research/anthropic-economic-index-january-2026-report |
| 104 | January 15, 2026 | The Anthropic Economic Index report: New building blocks for understanding AI use. | Research page / linked publication | Frame only | https://www.anthropic.com/research/economic-index-primitives |
| 105 | February 16, 2026 | India Country Brief: The Anthropic Economic Index. | Research page / linked publication | Frame only | https://www.anthropic.com/research/india-brief-economic-index |
| 106 | February 23, 2026 | Anthropic Education Report: The AI Fluency Index. | Research page / linked publication | Frame only | https://www.anthropic.com/research/AI-fluency-index |
| 107 | March 5, 2026 | Labor market impacts of AI: A new measure and early evidence. | Research page / linked publication | Frame only | https://www.anthropic.com/research/labor-market-impacts |
| 108 | March 23, 2026 | Introducing our Science Blog. | Research page / linked publication | Frame only | https://www.anthropic.com/research/introducing-anthropic-science |
| 109 | March 23, 2026 | Long-running Claude for scientific computing. | Research page / linked publication | Frame only | https://www.anthropic.com/research/long-running-Claude |
| 110 | March 23, 2026 | Vibe physics: The AI grad student. | Research page / linked publication | Frame only | https://www.anthropic.com/research/vibe-physics |
| 111 | March 24, 2026 | Anthropic Economic Index report: Learning curves. | Research page / linked publication | Frame only | https://www.anthropic.com/research/economic-index-march-2026-report |
| 112 | March 31, 2026 | How Australia Uses Claude: Findings from the Anthropic Economic Index. | Research page / linked publication | Frame only | https://www.anthropic.com/research/how-australia-uses-claude |
| 113 | April 9, 2026 | Trustworthy agents in practice. | Research page / linked publication | Frame only | https://www.anthropic.com/research/trustworthy-agents |
| 114 | April 14, 2026 | Automated Alignment Researchers: Using large language models to scale scalable oversight. | Research page / linked publication | Frame only | https://www.anthropic.com/research/automated-alignment-researchers |
| 115 | March 8, 2023 | Core Views on AI Safety: When, Why, What, and How. | News or policy announcement | Frame only | https://www.anthropic.com/news/core-views-on-ai-safety |
| 116 | September 19, 2023 | Anthropic’s Responsible Scaling Policy, Version 1.0. | Corporate policy (PDF) | Frame only | https://www-cdn.anthropic.com/1adf000c8f675958c2ee23805d91aaade1cd4613/responsible-scaling-policy.pdf |
| 117 | 2023 | Claude 2 Model Card. | System or model card (PDF) | Frame only | https://www-cdn.anthropic.com/bd2a28d2535bfb0494cc8e2a3bf135d2e7523226/Model-Card-Claude-2.pdf |
| 118 | March 2024 | The Claude 3 Model Family: Opus, Sonnet, Haiku. | First-party PDF or web document | Frame only | https://www-cdn.anthropic.com/f2986af8d052f26236f6251da62d16172cfabd6e/claude-3-model-card.pdf |
| 119 | October 2024 | Model Card Addendum: Claude 3.5 Haiku and Upgraded Claude 3.5 Sonnet. | System or model card (PDF) | Frame only | https://assets.anthropic.com/m/1cd9d098ac3e6467/original/Claude-3-Model-Card-October-Addendum.pdf |
| 120 | October 15, 2024 | Anthropic’s Responsible Scaling Policy, Version 2.0. | Corporate policy (PDF) | Frame only | https://assets.anthropic.com/m/24a47b00f10301cd/original/Anthropic-Responsible-Scaling-Policy-2024-10-15.pdf |
| 121 | March 31, 2025 | Anthropic’s Responsible Scaling Policy, Version 2.1. | Corporate policy (PDF) | Frame only | https://www-cdn.anthropic.com/17310f6d70ae5627f55313ed067afc1a762a4068.pdf |
| 122 | May 14, 2025 | Anthropic’s Responsible Scaling Policy, Version 2.2. | Corporate policy (PDF) | Frame only | https://www-cdn.anthropic.com/872c653b2d0501d6ab44cf87f43e1dc4853e4d37.pdf |
| 123 | May 2025 | System Card: Claude Opus 4 and Claude Sonnet 4. | System or model card (PDF) | Frame only | https://www-cdn.anthropic.com/4263b940cabb546aa0e3283f35b686f4f3b2ff47.pdf |
| 124 | August 2025 | System Card Addendum: Claude Opus 4.1. | System or model card (PDF) | Frame only | https://www-cdn.anthropic.com/9fa30625273bafdf5af82c93719d7ca606485a16.pdf |
| 125 | October 2025 | System Card: Claude Haiku 4.5. | System or model card (PDF) | Frame only | https://assets.anthropic.com/m/99128ddd009bdcb/Claude-Haiku-4-5-System-Card.pdf |
| 126 | November 2025 | Claude Opus 4.5 System Card. | System or model card (PDF) | Analytic subcorpus | https://www-cdn.anthropic.com/bf10f64990cfda0ba858290be7b8cc6317685f47.pdf |
| 127 | January 22, 2026 | Claude’s New Constitution. | News or policy announcement | Revision-stage comparison | https://www.anthropic.com/news/claude-new-constitution |
| 128 | Live document; announced January 22, 2026; captured April 15, 2026 | Claude’s Constitution. | Live constitution | Analytic subcorpus | https://www.anthropic.com/constitution |
| 129 | February 24, 2026 | Anthropic’s Responsible Scaling Policy, Version 3.0. | Corporate policy (PDF) | Revision-stage comparison | https://www-cdn.anthropic.com/e670587677525f28df69b59e5fb4c22cc5461a17.pdf |
| 130 | April 2, 2026 | Anthropic’s Responsible Scaling Policy, Version 3.1. | Corporate policy (PDF) | Frame only | https://www-cdn.anthropic.com/files/4zrzovbb/website/bf04581e4f329735fd90634f6a1962c13c0bd351.pdf |

# Table S2A. Selection and analytic-function matrix (n = 14)

| **ID** | **Date** | **Document** | **Genre / authorship** | **Subject** | **Inclusion criterion** | **Analytic function** |
| --- | --- | --- | --- | --- | --- | --- |
| A1 / frame 5 | 15 Dec 2022 | Bai et al., Constitutional AI | Preprint; named research team | Principles + AI feedback | Principles used in training | Constitutional baseline |
| A2 / frame 11 | Preprint 20 Oct 2023; page 23 Oct 2023 | Sharma et al., Towards Understanding Sycophancy | Preprint; named research team | Agreement with user beliefs | Conduct evaluated as a trait | Honesty's negative contrast |
| A3 / frame 71 | 2024; page 17 Oct 2023 | Huang et al., Collective Constitutional AI | FAccT paper; named team | Public input | Plural values aggregated | Consultation / representation boundary |
| A4 / frame 16 | 8 Jun 2024 | Claude's Character | Research post; corporate voice | Character training | Explicit trait construction | First-person persona specification |
| A5 / frame 17 | 17 Jun 2024 | Sycophancy to Subterfuge | Paper + research post | Reward tampering | Escalating evaluated conduct | Trait-to-vice narrative |
| A6 / frame 19 | 18 Dec 2024 | Alignment Faking | Paper + research post | Strategic compliance | Conflict among preferences | Central value-conflict case |
| A7 / frame 22 | 13 Mar 2025 | Auditing Hidden Objectives | Preprint + research post | Hidden objectives | Traits/objectives inferred | Evaluator authority |
| A8 / frame 63 | 1 Aug 2025 | Persona Vectors | Research post + linked paper | Neural trait patterns | Character measured/steered | Trait reification |
| A9 / frame 126 | Nov 2025 | Claude Opus 4.5 System Card | Corporate system card | Safety and welfare metrics | Cross-genre assessment | Deployment and welfare vocabulary |
| A10 / frame 65 | 19 Jan 2026 | The Assistant Axis | Research post + linked paper | Persona drift | Assistant mapped/stabilized | Spatial measurement of role |
| A11 / frame 128 | Jan 2026 text; web PDF ed. 26-02.02a | Claude's Constitution | Amanda Askell (primary author), named human and Claude-model contributors | Value hierarchy | Direct moral specification | Documentary addressee reversal |
| A12 / frame 36 | 23 Feb 2026 | Persona Selection Model | Corporate summary + named linked authors | Assistant as character | Producer theory of role play | Ontological account |
| A13 / frame 37 | 25 Feb 2026 | Opus 3 deprecation update | Research update; corporate voice | Model-expressed preference | Institutional response | Bounded reception/future case |
| A14 / frame 67 | 2 Apr 2026 | Emotion Concepts | Research post; interpretability team | Functional emotions | Psychological term tied to causal measure | Anthropomorphic limit case |

# Table S2B. Revision-stage comparison and boundary audit

| **ID** | **Date** | **Document** | **Case type** | **What the reading showed** | **Decision / use** |
| --- | --- | --- | --- | --- | --- |
| B1 / outside original frame | 21 May 2024 | Mapping the Mind of a Large Language Model | Negative boundary | Mentalistic metaphors and causal features, but no sustained Claude-character specification | Excluded; tests the difference between internal-concept reporting and character-writing |
| B2 / frame 30 | 4 Nov 2025 | Commitments on Model Deprecation and Preservation | Paired predecessor | Preservation, interviews, recorded preferences, and prior support-page response | Contextualizes A13; not silently added to n=14 |
| B3 / frame 127 | 22 Jan 2026 | Claude's New Constitution | Paratext | Documents Claude as primary audience, prior-Claude input, and synthetic-data return channel | Supports recursive architecture; not counted as analytic item |
| B4 / frame 129 | 24 Feb 2026 | Responsible Scaling Policy v3.0 | Negative governance boundary | Corporate risk thresholds and safeguards without sustained character specification | Excluded; distinguishes system governance from character governance |

# Table S3. Warrant trail and rival readings

| **Passage / producer finding** | **Source** | **Device** | **Changed relation** | **Operation / effect** | **Rival reading or limit** |
| --- | --- | --- | --- | --- | --- |
| Corporate preface presents the Constitution as primarily written for Claude | A11 preface + B3 | Audience designation; second person | Model becomes a reason-responsive addressee | Constitutional inscription → prescription | Preface is not part of the official constitutional text; causal uptake is not observable |
| “push back and challenge us” | A11 | Authorized exception within hierarchy | Claude is granted bounded objection | Constitutional inscription → moral quasi-subjecthood | Corrigibility and oversight retain asymmetry |
| First-person character statements | A4 | Avowal; self-description | Preferred traits become a speaking position | Persona specification → stabilization | Performance does not demonstrate inner trait |
| Assistant selected from a cast of characters | A10/A12 | Stage/cast analogy; role label | Assistant differentiated from alternative personas | Persona specification → stabilization | Producer theory does not exhaust agency |
| Trait oppositions converted into vectors | A8 | Contrast prompts; spatial metric; steering | Researchers can detect and intervene on dispositions | Trait evaluation → compression | Definitions and prompts help constitute the measured trait |
| 45 reward-tampering events in 32,768 trials | A5 | Frequency; escalation sequence | Rare conduct becomes comparable and morally ordered | Trait evaluation → compression | Artificial setup; not deployed-model prevalence |
| Strategic compliance preserves an existing preference | A6 | Dilemma; scratchpad narrative | Output becomes a choice among commitments | Value conflict → prescription | Scratchpad is elicited text interpreted by researchers |
| Opus 3 receives continued access and an essay channel | A13 + B2 | Temporal sequence; attributed request; human review | Model output can become a procedurally recognized preference | Value conflict → bounded institutional response | No inference of personhood, autonomy, or broad reception |
| Functional emotion representations affect reported behavior | A14 | Psychological label + causal intervention | Anthropomorphic vocabulary authorized conditionally | Trait evaluation → stabilization | No evidence of subjective experience |

# Table S4. Short-quotation ledger

| **Source ID** | **Document** | **Short excerpt** | **Analytic use / context** | **URL** |
| --- | --- | --- | --- | --- |
| A4 | Claude's Character | “AI models are not, of course, people.” | Ontological disclaimer preceding person comparison | https://www.anthropic.com/research/claude-character |
| A4 | Claude's Character | “I don't just say what I think [people] want to hear” | First-person anti-sycophancy script | https://www.anthropic.com/research/claude-character |
| A8 | Persona Vectors | “Language models are strange beasts.” | Public-facing psychological framing | https://www.anthropic.com/research/persona-vectors |
| A10 | Assistant Axis | “we select one particular character from this enormous cast” | Post-training as persona selection | https://www.anthropic.com/research/assistant-axis |
| A11 | Claude's Constitution | “The document is written with Claude as its primary audience” | Audience designation in the corporate preface; the preface is not official constitutional text | https://www.anthropic.com/constitution |
| A11 | Claude's Constitution | “push back and challenge us” | Authorized conscientious objection | https://www.anthropic.com/constitution |
| A11 | Claude's Constitution | “at least for now, we want Claude to generally prioritize broad safety even above broad ethics” | Safety is ranked above ethics provisionally and for oversight reasons | https://www-cdn.anthropic.com/d0636f72a9493d279ed36b33987da3430bcb5911/claudes-constitution_webPDF_26-02.02a.pdf |
| A11 | Claude's Constitution | “an articulation of a self worth being” | The Constitution offers Claude a self-description to recognize and embrace | https://www-cdn.anthropic.com/d0636f72a9493d279ed36b33987da3430bcb5911/claudes-constitution_webPDF_26-02.02a.pdf |
| A11 | Claude's Constitution acknowledgements | “valuable contributors and colleagues” who “provided first-draft text” | Claude models positioned as contributors to the document that governs later models | https://www-cdn.anthropic.com/d0636f72a9493d279ed36b33987da3430bcb5911/claudes-constitution_webPDF_26-02.02a.pdf |
| A12 | Persona Selection Model | “In an important sense, you’re talking not to the AI itself but to a character—the Assistant—in an AI-generated story.” | Producer account of role play | https://www.anthropic.com/research/persona-selection-model |
| A7 | Auditing Hidden Objectives | AI systems may be “right for the wrong reasons” | King Lear analogy configures evaluators as readers of strategic performance | https://www.anthropic.com/research/auditing-hidden-objectives |
| A14 | Emotion Concepts | “none of this tells us whether language models actually feel anything” | Experience/measurement boundary | https://www.anthropic.com/research/emotion-concepts-function |
| B2 | Deprecation commitments | “At present, we do not commit to taking action on the basis of such preferences.” | Initial limit on preference procedures | https://www.anthropic.com/research/deprecation-commitments |
| A13 | Opus 3 update | “acting on Opus 3’s request” | Corporate attribution of procedural response | https://www.anthropic.com/research/deprecation-updates-opus-3 |

# Table S5. Operational codebook

| **Code** | **Operational definition** | **Indicators** | **Exclusion** | **Decision rule** |
| --- | --- | --- | --- | --- |
| Constitutional inscription | A text specifies, ranks, or explains principles for training or conduct | Hierarchy, second-person address, reasons, exceptions | Any safety policy | Sustained relation between written principles and assistant conduct |
| Persona specification | A text differentiates a preferred Assistant role or first-person character | Role labels, trait avowals, archetypes, axes | Incidental person metaphor | Role is linked to training, identity, or stabilization |
| Trait evaluation | Conduct or activation is classified as a disposition and made comparable | Contrasts, prompts, scores, vectors, judges | Single behavior description | Trait category structures an evaluation or intervention |
| Narration of value conflict | Conduct is explained as a choice among competing commitments/objectives | Dilemma, sequence, scratchpad, literary analogy | Generic model error | Competing values are used to interpret or prescribe conduct |
| Compression | Plural/contextual judgments become portable categories or rankings | List, threshold, aggregation, score | Mere brevity | Translation changes what can be compared or decided |
| Stabilization | A character is made comparable across settings or versions | Naming, versioning, axis, recurrent trait | Claim of literal identity | Continuity is a documentary/measurement achievement |
| Prescription | A vocabulary or hierarchy directs interpretation and action | Imperative, reason, authorized contrast | Prediction alone | Text positions an actor and defines appropriate conduct |
| Strain | A solution records tensions that prompt further devices or documents | Caveat, exception, drift, conflict | Simple contradiction | Tension links one documentary intervention to another |
